# Emergency Medicine Residents Consistently Rate Themselves Higher than Attending Assessments on ACGME Milestones

**DOI:** 10.5811/westjem.2015.8.27247

**Published:** 2015-11-12

**Authors:** Katja Goldflam, Jessica Bod, David Della-Giustina, Alina Tsyrulnik

**Affiliations:** Yale University School of Medicine, Department of Emergency Medicine, New Haven, Connecticut

## Abstract

**Introduction:**

In 2012 the Accreditation Council for Graduate Medical Education (ACGME) introduced the Next Accreditation System (NAS), which implemented milestones to assess the competency of residents and fellows. While attending evaluation and feedback is crucial for resident development, perhaps equally important is a resident’s self-assessment. If a resident does not accurately self-assess, clinical and professional progress may be compromised. The objective of our study was to compare emergency medicine (EM) resident milestone evaluation by EM faculty with the same resident’s self-assessment.

**Methods:**

This is an observational, cross-sectional study that was performed at an academic, four-year EM residency program. Twenty-five randomly chosen residents completed milestone self-assessment using eight ACGME sub-competencies deemed by residency leadership as representative of core EM principles. These residents were also evaluated by 20 faculty members. The milestone levels were evaluated on a nine-point scale. We calculated the average difference between resident self-ratings and faculty ratings, and used sample t-tests to determine statistical significance of the difference in scores.

**Results:**

Eighteen residents evaluated themselves. Each resident was assessed by an average of 16 attendings (min=10, max=20). Residents gave themselves statistically significant higher milestone ratings than attendings did for each sub-competency examined (p<0.0001).

**Conclusion:**

Residents over-estimated their abilities in every sub-competency assessed. This underscores the importance of feedback and assessment transparency. More attention needs to be paid to methods by which residency leadership can make residents’ self-perception of their clinical ability more congruent with that of their teachers and evaluators. The major limitation of our study is small sample size of both residents and attendings.

## INTRODUCTION

In 2012 the Accreditation Council for Graduate Medical Education (ACGME) introduced the Next Accreditation System (NAS), which implemented milestones to assess the abilities and progress of residents. Each milestone is a significant, progressive, competency-based point in the development of a resident. These milestones evaluate accomplishments that identify specialty-specific knowledge, skills, attitudes and behaviors that can be used as outcome measures within the general competencies.^1^,^2^ Emergency medicine (EM) has developed 23 sub-competencies, with five milestone levels within each. Residents are expected to progress through levels of proficiency as they complete their training.^2^,^3^

Attending evaluation and feedback is crucial for resident development. However, at least as equally important is a resident’s self-assessment. This is because feedback from others is often interpreted and integrated through the framework of a learner’s self-assessment.^4^,^5^ Learners use an amalgam of self-assessment and feedback to generate actionable goals for improvement.^6^ If a resident does not accurately self-assess, clinical and professional progress may be compromised. A resident who is unable to accurately judge his or her own abilities may fail to achieve the necessary skills to be a safe and effective physician. In other words, failure to acknowledge deficiencies may lead to a failure to correct them.

Attending physicians working at academic centers are used as the benchmark in assessing a resident’s abilities as a physician. To date, no study has compared resident self-assessment to attending assessment using the standardized framework of the ACGME milestones.

### Goals of this Investigation

Our study used the framework of the ACGME milestones to compare EM resident evaluation by EM faculty with the same residents’ self-assessments.

## METHODS

This study is an observational, cross-sectional study performed at an academic EM residency. A human investigation committee (HIC) exemption was granted by the institutional IRB. All residents from EM post graduate year (PGY) 1 through 4 level were included in the study, with the exception of the single resident who helped to conduct the study. Twenty-five residents were chosen using a random number generator to participate in the study. The remaining residents were omitted due to time limitations on attendings filling out the forms and concerns that too large a number of evaluations would be prohibitive to attending willingness to participate in the study.

These residents completed self-assessments of milestone levels using eight ACGME sub-competencies that were chosen as representative of core EM principles by residency leadership consensus. Moreover, residency leadership agreed that a large group of attending evaluators would likely be able to comment on these, more familiar, sub-competencies for the majority of residents. The residency leadership consensus consisted of the residency program director and associate program directors. These included Emergency Stabilization (PC1), History and Physical (PC2), Diagnostic Studies (PC3), Diagnosis (PC4), Disposition (PC7), Communication (ICS1), Multi-Tasking (PC8), and Team Management (ICS2). These same residents were also evaluated by 20 faculty members using identical milestones. Faculty members have contact with residents in various settings, which include clinical shifts, simulation laboratory, and in small-group teaching sessions. Faculty members were able to opt out of assessing any resident whom they felt they could not evaluate due to limited interaction. The sub-competencies were evaluated on a nine-point scale, which reflects the rubric published by the ACGME (Figure). No advanced training or instruction was provided regarding the utilization of the ACGME milestones. No other evaluation tools were provided to faculty when they were asked to assign a score.

We calculated the average difference between resident self-ratings and faculty ratings. Sample t-tests were used to determine the statistical significance of the difference in scores. We carried out mixed models analyses to determine if there were any significant interactions between the rater type (self vs. attending) and program year. For each program year, we calculated and compared the difference in the least square means between residents and their attending raters to the overall difference in least square means for each sub-competency.

## RESULTS

Eighteen of the 25 residents surveyed completed the evaluation. Each resident was assessed by an average of 16 attendings (min=10, max=20). Residents gave themselves higher milestone ratings than attendings did for each of the eight sub-competencies evaluated (Table 1). The mean difference in score for each sub-competency was close to one point, with the exception of “Team Management,” which was 0.5 points. For seven out of eight sub-competencies, the difference in resident milestone self-assessment score and attending milestone assessment score was statistically significant (*p*<0.05)*.* The one sub-competency where statistical significance was not reached was “Team Management” (p=0.09).

Mixed model analysis showed statistically significant differences between self-ratings and attending ratings in most sub-competencies for the PGY 1 and 3 cohorts (Table 2 and Table 3). The PGY 2 cohort had fewer differences across sub-competencies, with statistically significant differences in only three sub-competencies (Table 4). For PGY 4, self and attending ratings did not significantly differ in any sub-competency (Table 5).

## DISCUSSION

Our study found that residents (combined PGY1 through PGY4) consistently rated themselves as more proficient for each sub-competency than did their attending evaluators. This is consistent with prior data showing that physician self-assessment typically does not correlate with external measures of performance.^7^ Although self-assessment may be inaccurate, it is important for evaluators to consider learner self-image when giving feedback. This feedback will undoubtedly be interpreted by the learner through a filter of his/her own perception.^8^ For example, feedback from an attending that is lower than a learner feels he/she attained, may be rejected by that learner who believes he or she has reached a higher level of proficiency. This could negatively impact the development and growth of that learner.

Our study illustrates that milestone-based assessment remains subject to these considerations. This suggests that educators must be cognizant of residents’ self-assessments when formulating and delivering feedback. Our subgroup analysis included small sample sizes; more work with larger sample sizes is necessary to determine if program year does indeed have an effect on agreement between resident and attending assessment. Within this context, our data showed that differences between self-assessment and attending assessment may be affected by program year. Unlike the results for PGY 1 through 3, self and attending ratings for PGY 4 did not differ significantly on any sub-competency. These results suggest that in PGY 4, self and attending ratings converge and are quite similar. It is important to note that these *p* values were not adjusted for multiple comparisons and should therefore be interpreted as only part of further exploratory analyses.

Taking the results of our study into consideration, the finding that residents perceive themselves as more capable than they are rated by attendings would be relevant to discussions in Clinical Competency Committee (CCC) meetings. Residents’ perception of their skills would be important in grading them on sub-competencies that deal with “practice-based learning and improvement.” Although difficult to put into practice, perhaps resident self-evaluations should be included in their “residency portfolios” and compared to the CCC rating of that resident to ensure that as the resident moves through the program self-perception is not significantly different from that of his/her evaluators.

## LIMITATIONS

The major limitation of this study is the small sample size of attendings and residents evaluated. Our self-assessment response rate was 70%. Self-assessment was not compulsory, as participation in research was voluntary per our HIC. It is not known if those who did not respond were different demographically or in PGY year, as the study personnel was blinded to the identities of the residents assessed. Another limitation is the varying levels of familiarity with the milestones among the residents and attendings surveyed. In addition, due to the nature of EM shift work, attendings have different frequencies of interactions with residents, which may introduce bias into their assessments. As the study was done at an academic institution, some faculty members do have less clinical time in the department than others. Although faculty members were permitted to opt out of assessing a resident with whom they had limited experience, we acknowledge that the frequency and types of faculty-resident interactions assessed may vary widely. In addition, the residents studied were at different levels of their training; this may have influenced their self-ratings. For example, some of the residents surveyed were close to graduation, a circumstance that may inflate their self-assessments. Although our data suggest that PGY 4 residents’ and attending evaluations may be similar, our interpretation is limited by the small number of representatives in each class; thus, more investigation is required to determine if there is a difference between classes in their ability to self-assess accurately. A larger sample of residents assessed may allow for more detailed sub-group analysis by PGY year. In addition, a larger sample size would also allow for more detailed analysis of high and low performers and their ability to self-assess, as had been demonstrated in the past. This study relies on the assumption that attending ratings are more accurate than resident self-rating, the validity of which may need further investigation.^4^,^7^ Perhaps most importantly, milestones are a relatively new assessment tool with very few studies evaluating their validity.^1^,^3^,^9^

## CONCLUSION

Residents over-estimated their abilities in each of eight sub-competencies assessed. This underscores the importance of feedback and assessment transparency. More attention needs to be paid to methods by which residency leadership can make residents’ clinical ability self-perception more congruent with that of their teachers and evaluators.

## Figures and Tables

**Figure:**
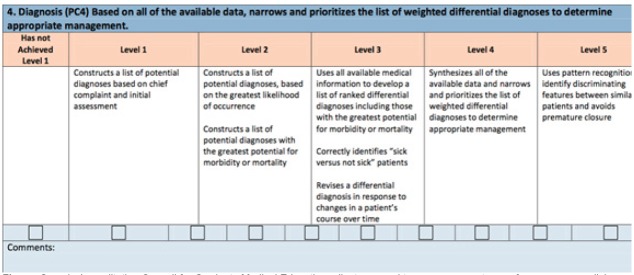
Sample Accreditation Council for Graduate Medical Education milestone used to assess competency of emergency medicine residents and fellows.

**Table 1:** Comparison of all residents’ post graduate years 1–4 self-rating to attending rating.

Evaluation construct	Self	Rater(s)	Estimated difference ± standard error	95% CL	*p*
Communication	6.68 ± 0.33	5.54 ± 0.10	1.14 ± 0.32	(0.49, 1.78)	0.0006
Diagnosis	6.77 ± 0.30	5.60 ± 0.56	1.16 ± 0.31	(0.08, 2.34)	0.0002
Diagnostic studies	6.89 ± 0.29	5.62 ± 0.08	1.26 ± 0.29	(0.68, 1.84)	<0.0001
Disposition	6.54 ± 0.31	5.52 ± 0.09	1.01 ± 0.31	(0.39, 1.63)	0.0015
Emergency stabilization	6.22 ± 0.30	5.51 ± 0.08	0.70 ± 0.30	(0.10, 1.30)	0.0212
History and physical	6.95 ± 0.33	5.72 ± 0.08	1.23 ± 0.33	(0.57, 1.89)	0.0003
Multi-tasking	6.80 ± 0.33	5.48 ± 0.08	1.31 ± 0.33	(0.65, 1.97)	0.0001
Team management	5.99 ± 0.31	5.47 ± 0.10	0.52 ± 0.30	(−0.08, 1.13)	0.0902

**Table 2:** Comparison of post graduate year 1 self-rating to attending rating.

Evaluation construct	Self	Rater(s)	Estimated difference ± standard error	95% CL	*p*
Communication	6.16 ± 0.60	4.14 ± 0.21	2.01 ± 0.60	(0.81, 3.21)	0.001
Diagnosis	5.17 ± 0.56	3.95 ± 0.16	1.21 ± 0.57	(0.09, 2.34)	0.0348
Diagnostic studies	6.56 ± 0.54	4.03 ± 0.17	2.52 ± 0.55	(1.45, 3.60)	<0.0001
Disposition	5.35 ± 0.58	3.92 ± 0.19	1.42 ± 0.58	(0.27, 2.58)	0.0152
Emergency stabilization	4.95 ± 0.56	3.69 ± 0.18	1.25 ± 0.56	(0.14, 2.36)	0.0265
History & physical	6.56 ± 0.61	4.12 ± 0.18	2.44 ± 0.62	(1.21, 3.66)	0.0001
Multi-tasking	5.17 ± 0.61	3.77 ± 0.17	1.39 ± 0.62	(0.17, 2.62)	0.0254
Team management	4.92 ± 0.57	4.07 ± 0.21	0.84 ± 0.57	(−0.27, 1.97)	0.1392

**Table 3:** Comparison of post graduate year 3 self-rating to attending rating.

Evaluation construct	Self	Rater(s)	Estimated difference ± standard error	95% CL	*p*
Communication	6.84 ± 0.55	5.79 ± 0.18	1.05 ± 0.55	(−0.03, 2.13)	0.0573
Diagnosis	7.50 ± 0.51	5.91 ± 0.14	1.59 ± 0.52	(0.56, 2.61)	0.0024
Diagnostic studies	7.17 ± 0.49	5.98 ± 0.15	1.19 ± 0.49	(0.21, 2.16)	0.0165
Disposition	6.51 ± 0.53	5.87 ± 0.16	0.63 ± 0.53	(−0.40, 1.68)	0.2291
Emergency stabilization	6.99 ± 0.51	5.89 ± 0.16	1.10 ± 0.51	(0.10, 2.11)	0.0311
History & physical	7.66 ± 0.55	6.08 ± 0.15	1.58 ± 0.56	(0.47, 2.68)	0.0053
Multi-tasking	7.49 ± 0.55	5.72 ± 0.15	1.76 ± 0.56	(0.65, 2.87)	0.0019
Team management	6.31 ± 0.52	5.68 ± 0.18	0.62 ± 0.51	(−0.39, 1.64)	0.2272

**Table 4:** Comparison of post graduate year 2 self-rating to attending rating.

Evaluation construct	Self	Rater(s)	Estimated difference ± standard error	95% CL	*P*
Communication	6.14 ± 0.67	5.07 ± 0.20	1.07 ± 0.67	(−0.24, 2.39)	0.1113
Diagnosis	6.70 ± 0.63	5.19 ± 0.15	1.50 ± 0.63	(0.25, 2.75)	0.0181
Diagnostic studies	6.43 ± 0.60	5.15 ± 0.16	1.28 ± 0.60	(0.09, 2.46)	0.0346
Disposition	6.91 ± 0.65	5.07 ± 0.18	1.83 ± 0.64	(0.56, 3.11)	0.0048
Emergency stabilization	5.87 ± 0.62	5.07 ± 0.17	0.79 ± 0.62	(−0.43, 2.02)	0.2043
History & physical	5.92 ± 0.68	5.20 ± 0.17	0.72 ± 0.68	(−0.62, 2.07)	0.2934
Multi-tasking	6.19 ± 0.68	5.10 ± 0.16	1.08 ± 0.68	(−0.26, 2.44)	0.1153
Team management	6.12 ± 0.64	5.03 ± 0.20	1.09 ± 0.63	(−0.15, 2.33)	0.0863

**Table 5:** Comparison of post graduate year 4 self-rating to attending rating.

Evaluation construct	Self	Rater(s)	Estimated difference ± standard error	95% CL	*P*
Communication	7.58 ± 0.78	7.15 ± 0.19	0.43 ± 0.77	(−1.09, 1.95)	0.58
Diagnosis	7.71 ± 0.72	7.35 ± 0.15	0.35 ± 0.73	(−1.08, 1.79)	0.626
Diagnostic studies	7.39 ± 0.69	7.34 ± 0.16	0.04 ± 0.69	(−1.31, 1.41)	0.9434
Disposition	7.38 ± 0.74	7.23 ± 0.18	0.15 ± 0.74	(−1.31, 1.62)	0.8399
Emergency stabilization	7.07 ± 0.72	7.40 ± 0.17	−0.33 ± 0.71	(−1.74, 1.08)	0.643
History & physical	7.67 ± 0.78	7.47 ± 0.16	0.19 ± 0.79	(−1.36, 1.75)	0.8054
Multi-tasking	8.33 ± 0.78	7.32 ± 0.16	1.01 ± 0.79	(−0.54, 2.57)	0.203
Team management	6.59 ± 0.73	7.06 ± 0.20	−0.46 ± 0.73	(−1.90, 0.97)	0.5271

## References

[1] Beeson MS, Carter WA, Christopher TA (2013). The development of the emergency medicine milestones. Acad Emerg Med.

[2] Nasca TJ, Philibert I, Brigham T (2012). The next accreditation system rationale and benefits. N Engl J Med.

[3] Korte RC, Beeson MS, Russ CM, Reisdorff EJ, Emergency Medicine Milestones Working Group (2013). The emergency medicine milestones: a validation study. Acad Emerg Med.

[4] Veloski J, Boex JR, Grasberger MJ (2006). Systematic review of the literature on assessment, feedback and physicians’ clinical performance: BEME Guide No. 7. Med Teach.

[5] Sargeant J, Armson H, Chesluk B (2010). The processes and dimensions of informed self-assessment. Acad Med.

[6] Bounds R, Bush C, Aghera A, MERC at CORD Feedback Study Group (2013). Emergency medicine residents’ self-assessments play a critical role when receiving feedback. Acad Emerg Med.

[7] Davis DA, Mazmanian PE, Fordis M (2006). Accuracy of physician self-assessment compared with observed measures of competence. JAMA.

[8] Eva KW, Armson H, Holmboe E (2012). Factors influencing the responsiveness to feedback: on the interplay between fear, confidence, and reasoning processes. Adv Health Sci Educ Theory Pract.

[9] Peck TC, Dubosh N, Rosen C (2014). Practicing emergency physicians report performing well on most emergency medicine milestones. J Emerg Med.

